# Failure rates of miniscrews inserted in the maxillary tuberosity

**DOI:** 10.1590/2177-6709.24.5.046-051.oar

**Published:** 2019

**Authors:** Muhammad Azeem, Arfan Ul Haq, Zubair Hassan Awaisi, Muhammad Mudassar Saleem, Muhammad Waheed Tahir, Ahmad Liaquat

**Affiliations:** 1 Faisalabad Medical University, Punjab Medical College - Dental Section, Department of Orthodontics (Faisalabad, Pakistan).; 2 De’Montmorency College of Dentistry, Department of Orthodontics (Lahore, Pakistan).; 3 Nishter Institute of Dentistry, Department of Orthodontics (Multan, Pakistan).; 4 Islamabad Medical & Dental College, Department of Oral and Maxillofacial Surgery (Islamabad, Pakistan).; 5 Allama Iqbal Medical College, Jinnah Hospital, Department of Oral and Maxillofacial Surgery (Lahore, Pakistan).; 6 University of Lahore, Department of Oral and Maxillofacial Surgery (Lahore, Pakistan).

**Keywords:** Tuberosity, Miniscrews, Failure.

## Abstract

**Introduction:**

Anchorage conservation in orthodontics has always been a challenge.

**Objective:**

The aim of this current study was to find out the failure rate of miniscrews inserted in the maxillary tuberosity (MT) region.

**Methods:**

This pilot study consisted of 40 patients (23 female, 17 male; mean age = 20.1±8.9 years) that had received 60 MT miniscrews for orthodontic treatment. Clinical notes and pictures were used to find out the primary outcome of miniscrew failure. Independent failure factors were also investigated. Logistic regression analysis was done for predictor’s relation with MT miniscrews failure.

**Results:**

There was no significant correlation in failure rate according to various predictor variables, except for miniscrews installed by lesser experienced operators, which showed significantly more failure. The odds ratio for miniscrew failure placed by inexperienced operators was 4.16.

**Conclusion:**

A 26.3% failure rate of mini-implants inserted in the MT region was observed.

## RESUMO

**Introdução:**

a manutenção da ancoragem sempre foi um desafio na Ortodontia.

**Objetivo:**

o objetivo do presente estudo foi descobrir a taxa de falhas dos mini-implantes instalados na região da tuberosidade maxilar (TM).

**Métodos:**

o presente estudo piloto avaliou 40 pacientes (23 mulheres, 17 homens; idade média = 20,1 ± 8,9 anos) que receberam 60 mini-implantes na TM durante o tratamento ortodôntico. Anotações clínicas e fotografias foram usadas para investigar o principal motivo para a falha do mini-implante. Fatores de insucesso independentes também foram investigados. Uma análise de regressão logística foi realizada para medir o impacto de cada fator preditivo sobre a falha na instalação dos mini-implantes na TM.

**Resultados:**

as diferentes variáveis preditivas não demonstraram correlação significativa com a taxa de falhas, com exceção da instalação dos mini-implantes realizada por operadores inexperientes, que mostrou quantidade significativamente maior de falhas. A razão de chances para a falha dos mini-implantes instalados por operadores inexperientes foi de 4,16.

**Conclusão:**

observou-se uma taxa de falhas de 26,3% para os mini-implantes instalados na região da TM.

## INTRODUCTION

Anchorage preservation has always been a challenging goal during orthodontic therapy,^1^ especially when simultaneous movement of group of teeth are planned. Extraoral appliances and intraoral elastics have conventionally been used, despite the compliance issue of extraoral bulky appliances,^2^ ^-^ ^3^ the adverse effects of anterior teeth extrusion and disruption of the occlusal plane from intraoral elastics.

Orthodontic anchorage preservation has always been a challenging goal, especially when movement of anterior teeth are planned in the presence of inadequate posterior dentition for orthodontic anchorage (e.g.: partial edentulism^4^), where one of the possible miniscrew insertion sites in those cases includes the maxillary tuberosity (MT).^5^ Even though the cortical bone is thin and density in the MT region is not ideal,^6^ the advantages of miniscrew placement at the MT region are: minimal risk of damage to molar roots and neurovasculature, which in turn expands the range of orthodontic tooth movement, especially for en-masse distalization and en-masse retraction of the maxillary teeth.^7^ ^-^ ^9^

Miniscrews can be labelled as successful if they remain functionally stable in jaw bones until the end of treatment or until intentional removal.^10^ Meanwhile, miniscrews are labelled as failed if they had any discernible mobility or had become loose during orthodontic treatment.^11^ ^,^ ^12^ There are various factors reported in literature that affect success and failure of miniscrews in the short and long term.^13^

Studies are present on the success rate of dental implants placed at MT region,^14^ but there is lack of literature regarding failure rate of miniscrews inserted in the MT area. Therefore, the aim of the current study was to investigate the failure rates of miniscrews inserted in the MT region, and to evaluate the associated factors. The null hypothesis was that failure rate of miniscrews inserted in the MT area are independent to the tested factors.

## MATERIAL AND METHODS

This retrospective pilot study was conducted after ethics approval of Faisalabad Medical University, and involved records of orthodontic patients from July 2012 to July 2018. Inclusion criteria were: All patients had received MT miniscrews (8 mm & 10mm long, 1.3 mm & 1.5mm in diameter),^15^ ^,^ ^16^ had complete orthodontic records, had updated record status of the miniscrews throughout the treatment in clinical notes, missing maxillary third molars, and had insignificant medical/drug history and non-smokers. Data of 40 patients (23 female, 17 male; mean age = 20.1±8.9 years) who had 60 MT miniscrews inserted and met the inclusion criteria, were included (Table 1). 

**Table 1:** Failure rates of MT miniscrews by features of orthodontic patients.

Predictor		n	Failure rate (%)
Age	<18 years	27	21.34
	>18 years	33	19.23
Sex	Male	26	24.32
	Female	34	21.54
Diameter of miniscrew	1.3 mm	37	21.34
	1.5 mm	23	25.26
Length of miniscrew	8 mm	41	22.64
	10 mm	19	26.22
Force amount	100 g	31	23.41
	> 100 g	29	24.04
Force delivery	Elastomerics	21	27.34
	NiTi coils	39	26.59
Oral hygiene	Poor	7	27.09
	Good	53	22.35
Operator experience	Experienced	27	2.41
	Inexperienced	33	42.09
Side of miniscrew	Left	28	21.53
	Right	32	28.04
Inflammation	Yes	8	25
	No	52	20.04

**Figure 1:**
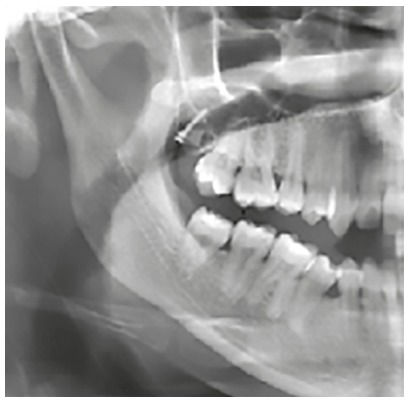
Miniscrew inserted in the maxillary tuberosity region.

All the miniscrews were placed by self-tapping method, under local anaesthesia at an angulation of 20-40 degrees to the occlusal plane vertically (Fig 1), by two types of operators: 27 miniscrews by an expert operator and 33 miniscrews by inexperienced post-graduate residents under supervision. All the MT miniscrews were used for the maxillary molar distalization, and immediate orthodontic loading (100-150 g) was applied from miniscrews by elastomerics or using nickel-titanium coil springs (12 mm). 

Based on the intraoral photographs and clinical file notes, subjects were divided into the following groups: good or poor oral hygiene, and presence or absence of inflammation. Clinical notes and photographs were used to find out the primary outcome of miniscrew failure, and independent failure factors were also investigated as predictors of miniscrew failure (Table 1). 

After insertion, primary stability was evaluated by an expert operator, with cotton tweezers. Miniscrews were labelled as successful if they remained functionally stable in MT until the end of treatment or until intentional removal, while miniscrews were labelled as failed if they had any discernible mobility or had become loose during orthodontic treatment.^10^ ^-^ ^12^ Vertical analysis was done using measurement of mandibular plane angle (MPA) at pre-treatment cephalograms.

### Statistical analysis

The primary outcome of MT miniscrew failure was analyzed as a binomial variable (Failure: yes/no), and simple descriptive statistics were applied to calculate the failure rates across various predictor factors (Table 1). Logistic regression analysis was used to predict the impact of each variable in relation to the outcome of MT miniscrew failure. The regression models were fit using generalized estimating equations method. Odds ratio (relative risk) was measured for each failure factor. 

## RESULTS

Analysis of predictor variables showed that there was insignificant correlation in failure rate according to various predictor variables, except for the experience of the operator, where the less-experienced operators showed significantly more miniscrew failure (Table 1). 

The results of the logistic regression analyses are shown in Table 2. The odds ratio for miniscrew failure placed by inexperienced operators was 4.16 (Table 3). The overall failure rate was 26.3.1% for MT miniscrews. Average mandibular plane angle (MPA) of sample was 41.3 ± 4.01^o^ at pre-treatment.

**Table 2:** Logistic regression analysis

Variable		Estimate	Odds ratio	p-value
Age	<18 years	−0.1221	0.83	0.321
	>18 years	Reference		
Sex	Female	−1.1856	0.21	0.421
	Male	Reference		
Diameter of miniscrew	1.3 mm	0.4213	1.72	0.111
	1.5 mm	Reference		
Length of miniscrew	8 mm	0.5123	1.63	0.578
	10 mm	Reference		
Force amount	100 g	-0.3165	0.64	0.934
	> 100 g	Reference		
Force delivery	Elastomerics	−0.4098	0.75	0.572
	NiTi coils	Reference		
Oral hygiene	Poor	1.0476	1.76	0.321
	Good	Reference		
Operator experience	Experienced	1.5	4.16	0.001
	Inexperienced	Reference		
Side of mini-implant	Right	-0.4333	1.62	0.123
	Left	Reference		
Inflammation	Yes	-0.3753	0.61	0.138
	No	Reference		

**Table 3:** Odds ratio for failure of MT miniscrews.

Factor	Estimate	Odds ratio	p-value	95% Confidence Interval (CI)
Inexperienced Operator	1.5	4.16	0.001	2.071-6.321

## DISCUSSION

Nakao et al.^7^ showed that for orthodontic anchorage, maxillary tuberosity is a good site for insertion of a miniscrew, if there is enough space for its insertion. A systematic review on the success rate of dental implants placed at MT region showed an overall survival rate of 94.63%,^14^ but there is lack of literature evidence regarding failure rate of orthodontic miniscrews inserted in the MT site. Therefore, the aim of the present study was to investigate the failure rates of miniscrews inserted in the MT region of the maxilla and to evaluate the associated factors. 

One key variable responsible for initial stability of miniscrews is vertical growth pattern. High angle subjects were found to have reduced cortical bone density and thickness, which may influence initial stability of the miniscrews.^17^ Low angle subjects were found to have increased cortical bone density and thickness than other vertical types.^18^ ^,^ ^19^ Moon et al^20^ found a failure rate (23%) for interradicular miniscrews in high angle patients similar to the failure rate (26%) for MT miniscrews in patients of the present study, in which sample the mean mandibular plane angle (MPA) was 41.3 ± 4.01^o^ at pre-treatment. 

In the present study, as all the miniscrews were inserted with the same technique, the influence of surgical factors on the failure of the miniscrews was not investigated, except the factor of operator experience - which was the only factor significantly associated with the failure of miniscrews in the present study. Miniscrews on the right side of the jaw had a insignificantly higher failure rate, which may be due to better hygiene on the left side of the dentition by right-handed patients, who present the majority of the population.^21^ ^,^ ^22^ Good oral hygiene may also reduce inflammation around the miniscrews. In this study, oral hygiene and inflammation did not affect the failure rate. 

In this study, the mean duration of force application to the miniscrews was 58 weeks, which covered the critical time period of 40-48 weeks^21^. Sung et al^16^ recommended using a relatively long miniscrew with a diameter of 1.3 - 1.5 mm in atypical sites like MT.Lee and Baek^23^ showed that miniscrews with a diameter of 1.5mm or more can cause greater trauma to the cortical bone, with a negative effect on alveolar bone remodeling and miniscrew stability. Therefore, we chose subjects having MT miniscrew with a diameter of 1.3 to 1.5 mm and a length of 8 to 10mm, which is in accordance with other available studies on MT miniscrews.^9^ ^,^ ^16^ ^,^ ^23^

The findings of the present study showed that MT miniscrews have lower success rate (73.7%) than that of the miniscrew inserted at other intraoral sites. This is in agreement with Venkateswaran et al,^8^ ^,^ ^9^ who found that MT miniscrews show comparatively high failure rates. A 21.8% failure rate of miniscrews inserted in the infrazygomatic area was found in recently conducted study,^24^ and another study found a 7% failure rate of miniscrews inserted in the extra-alveolar buccal shelf area.^25^ The median failure risk of palatal miniscrews was 6.1% in a recently conducted study.^26^ In a recently conducted systematic review, the overall failure rate of miniscrews was 13.5%.^27^

Although these were consecutively placed miniscrews, the main limitation of this study is its retrospective design, with associated risk of reporting and selection bias. Other limitations are sample size, lack of blinding, and the use of variable implants/conditions throughout the cases (variable lengths, diameters, etc). However, despite all these limitations, the present study provided literature for expected success rate of MT miniscrews and showed that in order to minimize the MT miniscrew failure, experienced clinicians should attempt its insertion. Further large scale prospective studies with improved methodology are suggested. 

## CONCLUSION

» A 26.3% failure rate of mini-implants inserted in the MT region was observed.
» Mini-implants were more successful when inserted in the MT region by experienced operators.
